# Vitreous cytokine levels following the administration of a single 0.19 mg fluocinolone acetonide (ILUVIEN®) implant in patients with refractory diabetic macular edema (DME)—results from the ILUVIT study

**DOI:** 10.1007/s00417-022-05564-2

**Published:** 2022-03-03

**Authors:** Svenja K. Deuchler, Ralf Schubert, Pankaj Singh, Adonis Chedid, Ninel Kenikstul, Julia Scott, Thomas Kohnen, Hanns Ackermann, Frank Koch

**Affiliations:** 1 Augenzentrum Frankfurt, Georg-Baumgarten-Str. 3, Frankfurt am Main, 60549 Germany; 2 Department of Ophthalmology, Goethe University Hospital, Frankfurt am Main, Germany; 3 Pneumological-Immunological Laboratory, Goethe University Hospital, Frankfurt am Main, Germany; 4 Institute of Biostatistics, Goethe University Hospital, Frankfurt am Main, Germany

**Keywords:** Cytokines, Diabetic macular edema, Diabetic retinopathy, Fluocinolone acetonide, Growth factors, Inflammatory markers

## Abstract

**Purpose:**

To investigate the changes in vitreous inflammatory and angiogenic cytokine levels, primarily interleukin-(IL)-6, following intravitreal injection of the 0.19 mg fluocinolone acetonide (FAc, ILUVIEN®) implant in patients with diabetic macular edema.

**Methods:**

A single-center phase IV study involving 12 patients’ eyes with diabetic macular edema. Vitreous fluid samples were obtained prior to intravitreal injection of the fluocinolone acetonide implant and then again over a 6-month period. Vitreous samples were examined using a cytometric bead array to measure IL-6, IL-8, IP-10, MCP-1, VEGF, and CD54. PIGF and PEDF were measured using an enzyme-linked immunosorbent assay. Changes in the cytokine and chemokine expression patterns were analyzed. Clinical parameters such as BCVA and center point thickness (CPT) were also examined.

**Results:**

There were mean reductions in all parameters between baseline and month 6. Significant changes (*p* < 0.05 versus baseline) were observed in the expression of IL-6, IP-10, MCP-1, and CD54 following the administration of fluocinolone acetonide implant. VEGF and PIGF increased at month 1 before declining at month 6, though this trend was not significant. CPT decreased rapidly between screening and the first follow-up visit, and this decrease was sustained. BCVA remained relatively stable throughout.

**Conclusion:**

This study demonstrated changes in vitreous inflammatory and angiogenic cytokine levels following intravitreal injection of the FAc implant in patients with diabetic macular edema. Data show that the fluocinolone acetonide implant led to rapid and sustained reductions of some inflammatory cytokines with improvement of the overall clinical picture.

**Supplementary Information:**

The online version contains supplementary material available at 10.1007/s00417-022-05564-2.


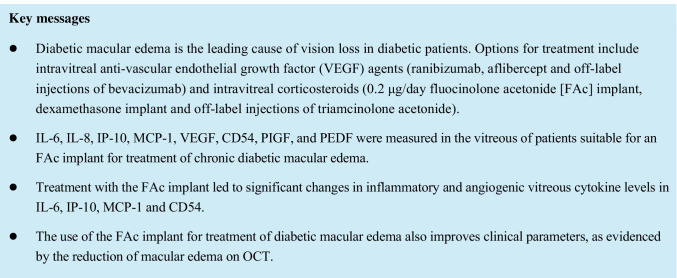


## Introduction

Diabetic macular edema (DME) is a complication of diabetic retinopathy (DR) and is the leading cause of vision loss in diabetics. It is estimated that between 14 and 25% of patients with diabetes develop DME within 10 years of being diagnosed and that the risk of development is influenced by both the type of diabetes and its duration [1].

Current treatment options for DME include intravitreal anti-vascular endothelial growth factor (VEGF) agents (ranibizumab, aflibercept and off-label injections of bevacizumab) and intravitreal corticosteroids (0.2 µg/day fluocinolone acetonide [FAc] implant, dexamethasone implant, and off-label injections of triamcinolone acetonide).

The FAc ILUVIEN® implant was designed to provide continuous treatment with fluocinolone acetonide for up to 3 years [2] and is delivered via an intravitreal injection for the treatment of DME that persists or recurs despite therapy. The outcomes from both clinical trials [3] and real-world practices [4, 5] have consistently shown that therapy with the FAc implant leads to rapid and sustained improvements of visual acuity and center point thickness (CPT).

Corticosteroids are multi-factorial in nature and target VEGF and several inflammatory cytokines relating to the development of DR and DME [6]. However, the actual effect of FAc on particular inflammatory cytokines still remains unclear due to limited research on humans in this area [7] and extrapolation of findings from other agents such as triamcinolone acetonide [8]. Previous investigations have shown that the pharmacokinetics of the FAc implant are unique compared to other commonly injected intravitreal corticosteroids as it delivers a lower daily dose of corticosteroid over a 3-year period [2], meaning that other preparations have a limited durability of release compared to the FAc implant [9].

Several cytokines have been documented to play a role in disease pathogenesis. VEGF is considered central to DME due to its pro-inflammatory, pro-angiogenic, and pro-vascular permeability effects [10]. The importance of VEGF in the development of DME is also reflected by the array of anti-VEGF therapies available and their use as first-line therapies. DME trials testing the effect of anti-VEGF agents have shown that around 40% of patients do not respond sufficiently to anti-VEGF agents, which suggests that other mediators play a role in the pathogenesis of DME [11]. In such cases, therapies that target inflammatory cytokines for example intercellular adhesion molecule-1 (CD54) and interleukin (IL)-6, may be a more effective therapeutic strategy [12]. The benefits of this approach seem to be reflected in clinical trials where it has been shown that corticosteroid agents, such as the FAc implant, are effective when used as a second-line therapy [4, 5].

A number of studies have investigated the effect of anti-VEGFs (ranibizumab [13, 14], aflibercept [15] and bevacizumab [8, 16]) on intraocular cytokine concentrations. There are no studies, however, that have investigated cytokine concentrations after the intravitreal injection of the FAc implant. The objective of this study was to determine its action on the expression of inflammatory and angiogenic cytokines, focusing on the change in factor IL-6 which plays a key role in the development of DME [6, 17–20] prior to and 6 months after the administration of the FAc implant in patients with DME that persisted or recurred despite treatment.

## Methods

### Ethics statement and patient recruitment

This pilot study was approved by the Bundesinstitut für Arzneimittel und Medizinprodukte (BfARM EudraCT-Number, 2016–004,488-38) and the ethics committee of the medical department of the Goethe University Frankfurt (E145/17). It was conducted at the Goethe University Hospital Frankfurt in Germany. The study was performed in accordance with the ethical standards as laid down in the 1964 Declaration of Helsinki and its later amendments. The study was designed as a phase IV trial involving 12 patients with DME that persisted or recurred despite treatment and therefore were eligible for treatment with the FAc implant. This was based on clinical judgment and decided prior to study initiation. Written informed consent was obtained from patients who wished to participate. Patient recruitment was from September 2018 to February 2019.

### Data collection

Initial data collection and screening took place 0–4 weeks prior to start of treatment with FAc implant. Patients were excluded if they (1) had a prior vitrectomy (3-port pars plana vitrectomy); (2) had a prior partial vitrectomy with drug administration; (3) had been treated with an anti-angiogenic agent; (4) had been treated with laser coagulation during the 90 days prior to screening; (5) had been treated with a long-acting corticosteroid during the 90 days prior to screening; (6) had undergone cataract extraction during the 90 days prior to screening; (7) had secondary cataract treatment in the 90 days prior to screening; (8) presented with other eye diseases at the time of screening; (9) had uncontrolled intraocular pressure (≥ 30 mmHg); (10) had uncontrolled hypertension (> 160/90 mmHg); (11) had poorly controlled diabetes (HbA1c > 10%); and (12) if they were previously diagnosed with an autoimmune disease.

All study participants had an indication for and planned implantation of FAc. Additional inclusion criteria included age ≥ 18 years old and a diagnosis of either type 1 or 2 diabetes mellitus with DR and DME. A best corrected visual acuity (BCVA) between 19 and 78 letters using the Early Treatment Diabetic Retinopathy Study (ETDRS) scoring system was required, as well as a CPT ≥ 250.

Diabetic disease status (date of first diagnosis, diabetes type, current medication, last HbA1c value) were collected at T-1. A blood sample (5–10 ml blood) was also taken to determine blood sugar and HbA1c levels. Other parameters that were assessed included disease and treatment history (which included the diagnoses of DR and DME), prior treatments (laser, intravitreal injections, vitrectomies) and relevant concomitant diseases. Optical coherence tomography (OCT) (Topcon 3D-OCT-2000) and fluorescein angiography (FA) (Zeiss FF 450 +) were also obtained at screening and were used to confirm presence of DME.

### Surgical procedure

A minimally invasive partial pars plana vitrectomy (cPPV) was performed at baseline (FAc implantation) to extract vitreous from the eye for lab analysis. A minimum volume of 0.5 ml of pure vitreous was aspirated before the infusion was activated and the pressure was regulated towards normal interoperative levels (see video, supporting information 1, which shows a limited 2-port pars plana vitrectomy with vitreous probe). This procedure has been reported previously [21] and was associated with minimal complications (0.44%) which were comparable to those following the administration of intravitreal medications. Vitreous fluid samples were obtained prior to intravitreal injection of the FAc implant and repeated at 1 month and 6 months after insertion.

### Primary and secondary parameters

Extracted vitreous samples were examined using a Cytometric Bead Array (CBA) and Flex sets [22–24] to determine IL-6, IL-8, IP-10 (pro-inflammatory cytokines), MCP-1 (pro-inflammatory chemokine), VEGF (growth factor), and CD54 concentrations (intercellular adhesion molecule). Placental growth factor (PIGF) and platelet-derived growth factor (PEDF) were measured using an enzyme-linked immunosorbent assay (ELISA; R&D Systems). The primary parameter in this study was the change in IL-6 and other cytokines were deemed secondary parameters.

Other (tertiary) parameters measured prior to and following intravitreal injection of the FAc implant included visual acuity (VA; measured using an ETDRS letter score), contrast VA (measured using a Central Vision Analyzer), CPT and average retinal thickness (ART) (both measured using OCT). Biomicroscopy was used to assess the lens and fundus state. FA was utilized to assess DR and color fundus photography was used to classify DR according to EDTRS. Finally, safety was determined from measurements of intraocular pressure (IOP) which was recorded during the day at both pre- and post-operative (i.e. post-PPV) time points. Post-operative measurements were taken 30 to 60 min following PPV.

### Statistical analysis

The Friedmann test was used to check whether there were differences in the position parameters between the samples. In some cases, a Student’s *t*-test was used to compare mean values between individual time points. A *p*-value < 0.05 was taken as representing a statistically significant difference. Variables were recorded at screening, baseline: the time the FAc implant was administered (first vitreous probe), 1 week after the implant: this time point was used for the recording of some of the study variables, 1 month after the FAc implant, second vitreous probe: 3 months after FAc implant, and 6 months after the FAc implant: third vitreous probe. These time points are presented throughout. Values are reported as mean ± standard deviation throughout unless otherwise stated.

## Results

Twelve patients underwent cPPV after screening. Thirteen patients were screened initially and assessed to be eligible to participate, but 1 declined to participate. Table 1 reports patient demographics and treatment history at screening for the 12 patients enrolled. The mean age of treated patients was 60.9 (range, 43–78) years. Eleven of the patients had type II diabetes and 3 of 12 study eyes were already pseudophakic at screening (Table 1). Prior therapies included laser with 4 patient eyes receiving panretinal laser coagulation, 2 receiving focal laser coagulation, and one receiving both focal and panretinal laser coagulation. Four patient eyes received panretinal cryocoagulation (Table 1). Intravitreal injections were given in 8 patient eyes (an average of 9.8 injections) with 6 eyes receiving aflibercept, 4 eyes receiving ranibizumab (7.0 injections) and 2 eyes receiving a dexamethasone implant (1.5 injections). Table 2 contains individual patient demographics and prior treatment details.

**Table 1:** Patient demographics and treatment history at time of screening

Parameter	Values (*n* = 12)
Mean age (range), years	60.9 (43–78)
Right/left eye, *n* (%)	6/6 (50.0/50.0)
Mean duration since diabetes diagnosis (range), years	13.8 (1–30.5)
Type-II diabetes, *n* (%) Type-I diabetes, *n* (%)	11 (91.7) 1 (8.3)
Mean HbA1c ± SD, %	7.0 ± 0.9
Mean duration since DR diagnosis (± SD), months	29.5 ± 26.2
Mean duration since DME diagnosis (± SD), months	19.8 ± 13.7
Phakic eyes, % (*n*)	25.0 (3)
Prior core vitrectomy, % (*n*)	41.7 (5)
Prior DME treatment	
Laser Coagulation, % (*n*)	91.7 (11)
Focal laser	18.2 (2)
Panretinal laser	36.4 (4)
Focal + Panretinal laser	9.1 (1)
Panretinal Cryocoagulation	36.4 (4)
Anti-VEGF intravitreal injections, % (mean # of injections)	66.7 (9.8)
Ranibizumab	33.3 (7.0)
Aflibercept	50.0 (8.3)
Bevacizumab	0.0 (0.0)
Intravitreal corticosteroids, % (mean # of injections)	16.7 (1.5)
Dexamethasone	16.7 (1.5)
Triamcinolone	0.0 (0.0)

**Table 2:** Individual patient demographics, screening data, and treatment history

Patient	Age and sex	Study eye	DM type	Years since diabetes diagnosis	Taking insulin	HbA1c at screening	Blood pressure at screening	EDTRS classification of diabetic retinopathy	Most recent treatment	Time since last treatment
ILV-01	49, male	Left	2	1.1	Yes	5.6	140/70	High-risk PDR	Limited PPV & Panretinal Cryocoagulation	101 days
ILV-02	60, male	Left	2	13.2	Yes	5.9	120/80	Severe NPDR	Aflibercept	97 days
ILV-03	56, male	Right	2	9.7	No	6.5	120/80	Moderate NPDR	Limited PPV	5 months
ILV-04	78, male	Left	2	10.2	Yes	6.6	120/70	Severe NPDR	Panretinal Laser	4 years
ILV-05	59, male	Right	2	29.3	Yes	8.5	140/70	Mild-moderate PDR	Panretinal Laser	94 days
ILV-06	60, male	Left	2	10.4	Yes	8.0	160/85	Very severe NPDR	Dexamethasone implant	97 days
ILV-07	70, female	Left	2	30.5	Yes	7.1	150/85	Moderate NPDR	Aflibercept	4 years
ILV-08	55, female	Right	2	1.0	Yes	6.7	160/80	Very severe NPDR	Panretinal Cryocoagulation	112 days
ILV-10	43, male	Right	1	20.5	Yes	7.2	120/90	High-risk PDR	Ranibizumab	9 months
ILV-11	77, male	Right	2	18.6	Yes	7.7	130/70	Very severe NPDR	Ranibizumab	1 year
ILV-12	64, male	Right	2	18.6	Yes	8.1	140/80	Severe NPDR	Aflibercept	112 days
ILV-13	60, male	Left	2	2.6	No	6.5	160/80	Very severe NPDR	Dexamethasone implant	11 months

Notes: Patient ILV-09 declined to participate in the study

### Volume of vitreous probes

In *n* = 23 cases, 1.2 ml volume could be extracted from the posterior compartment of the vitreous. In *n* = 13 cases, the minimum volume (0.5 ml) was reached with extraction volumes of 0.5 to 0.615 ml at baseline [*n* = 5], month 1 [*n* = 5], and month 6 [*n* = 3]).

### Changes in IL-6 (primary objective)

**Fig. 1:**
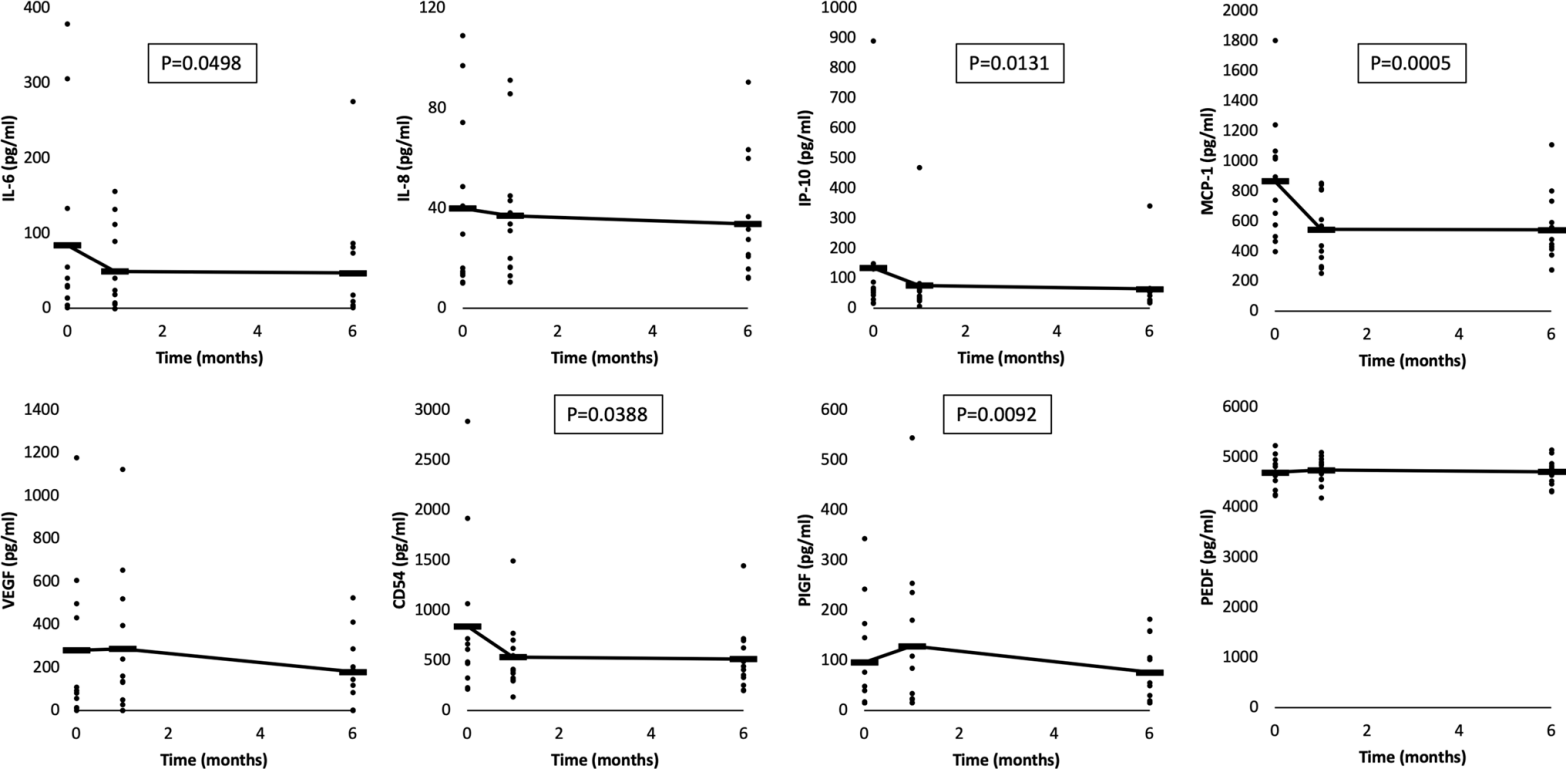
Vitreous inflammatory and angiogenic cytokine levels during therapy with FAc. Notes: Individual data plots are presented with mean values shown as a solid line. *P*-values reported where *P* < 0.05 and were calculated using a Friedmann test

One month after administration of the FAc implant, IL-6 decreased from 84.30 (± 126.81) pg/ml to 49.35 (± 57.07) pg/ml at month 1 and 46.93 (± 79.63) pg/ml at month 6 (see Table 3 and Fig. 1) (*p* = 0.0498).

**Table 3:** Cytokine changes baseline, and 1 month, and 6 months after treatment with the FAc implant

Cytokine	Baseline	1 month after implant	6 months after implant	6-months minus baseline	*P*-value*
IL-6	84.3 ± 126.8	49.3 ± 57.1	46.9 ± 79.6	-37.4 ± 83.8	*P* = 0.0498
IL-8	39.9 ± 35.3	37.1 ± 26.7	33.8 ± 25.0	-6.1 ± 25.7	*P* = 0.3679
IP-10	134.8 ± 241.7	76.5 ± 125.2	64.1 ± 88.9	-70.7 ± 155.3	*P* = 0.0131
CD54	839. ± 793.8	532.3 ± 354.7	513.8 ± 343.1	-325.6 ± 610.0	*P* = 0.0388
MCP-1	866.5 ± 401.0	545.6 ± 235.6	541.9 ± 241.0	-324.6 ± 305.5	*P* = 0.0005
VEGF	280.3 ± 351.5	286.4 ± 337.9	179.6 ± 164.0	-100.7 ± 313.0	*P* = 0.6136
PIGF	95.8 ± 108.4	128.5 ± 157.7	75.8 ± 63.2	-20.1 ± 67.7	*P* = 0.0092
PEDF	4696.2 ± 315.8	4743.0 ± 270.4	4711.5 ± 266.6	15.3 ± 439.6	*P* = 0.3385

Notes: Data presented as mean ± SD. Concentrations reported in pg/ml. * Friedmann test

### Changes in IL-8, IP-10, MCP-1, VEGF, CD54, PIGF, and PEDF (secondary objectives)

Following administration of the FAc implant there were significant overall changes in mean MCP-1 *(p* = 0.0005), CD54 (*p* = 0.0388), PIGF (*p* = 0.0092), and IP-10 (*p* = 0.0131) between baseline and month 6. In contrast there were no significant (*p* > 0.05) changes in IL-8, VEGF, or PEDF (Table 3).

### Visual acuity and contrast visual acuity outcomes (tertiary objectives)

At screening, mean visual acuity was 67.0 ± 9.3 ETDRS letters and remained stable at month 6 (66.4 ± 10.2 letters; *P* > 0.05, Student’s paired *t*-test). Contrast visual acuity also remained relatively stable with mean values of 29.5 ± 3.6 letters and 27.1 ± 3.3 letters (*P* > 0.05, Student’s paired *t*-test) at screening and at month 6. Table 4 shows individual patient results for visual acuity and contrast visual acuity.

**Table 4:** Individual patient OCT and visual acuity results

	**Center Point Thickness (µm)**					**Average Retinal** **Thickness (µm)**					**Best Corrected** **Visual Acuity (letters)**							**Best Corrected** **Contrast Visual Acuity (letters)**		
Patient	screening	1 week after implant	1 month after implant	3 months after implant	6 months after implant	screening	1 week after implant	1 month after implant	3 months after implant	6 months after implant	screening	baseline	1 week after implant	1 month after implant	3 months after implant	6 months after implant	screening		1 month after implant	6 months after implant
ILV1-01	368	285	253	Not measured	223	391	373	338.3	Not measured	292.7	77	77	83	76	Not measured	79	34		28	30
ILV1-02	467	441	441	446	330	362.9	349	337.5	344.3	304.8	73	73	78	79	75	73	30		25	22
ILV1-03	351	344	325	323	309	329	326.3	312.8	300.9	290.1	75	75	73	78	78	57	31		31	32
ILV1-04	383	348	338	338	354	294.5	294.3	290.6	284.2	280.5	61	62	64	66	70	67	29		29	29
ILV1-05	566	422	399	365	413	400.6	383.5	369.7	343.6	363.1	71	71	74	69	71	69	34		34	30
ILV1-06	236	237	241	169	275	365.2	363.1	339.9	347.8	370.2	47	47	51	47	48	43	35		31	29
ILV1-07	266	259	249	234	215	274.9	271.2	275.4	272.3	266.4	66	69	69	68	70	72	28		29	23
ILV1-08	317	209	174	209	191	383.2	338.5	322.5	325.3	315.2	64	64	65	64	68	68	25		25	28
ILV1-10	492	282	301	452	487	404.4	347.1	348.4	384.8	431.1	77	76	78	84	80	76	24		25	26
ILV1-11	564	314	381	318	271	444.6	348	350.1	335.4	316.8	74	74	70	69	72	69	26		28	27
ILV1-12	353	273	239	241	241	298.1	282	270.7	270.2	267.6	56	56	64	54	53	54	28		27	27
ILV1-13	599	526	506	508	576	425.6	369.6	376.6	376.6	401.7	63	63	67	73	73	70	30		30	22
Mean	413.5	328.3	320.6	327.5	323.8	364.5	337.13	327.7	325.9	325.0	67	67.3	69.7	68.9	63.2	66.4	29.5		28.5	27.1

Notes: Patient ILV-09 declined to participate in the study

### OCT outcomes

**Fig. 2:**
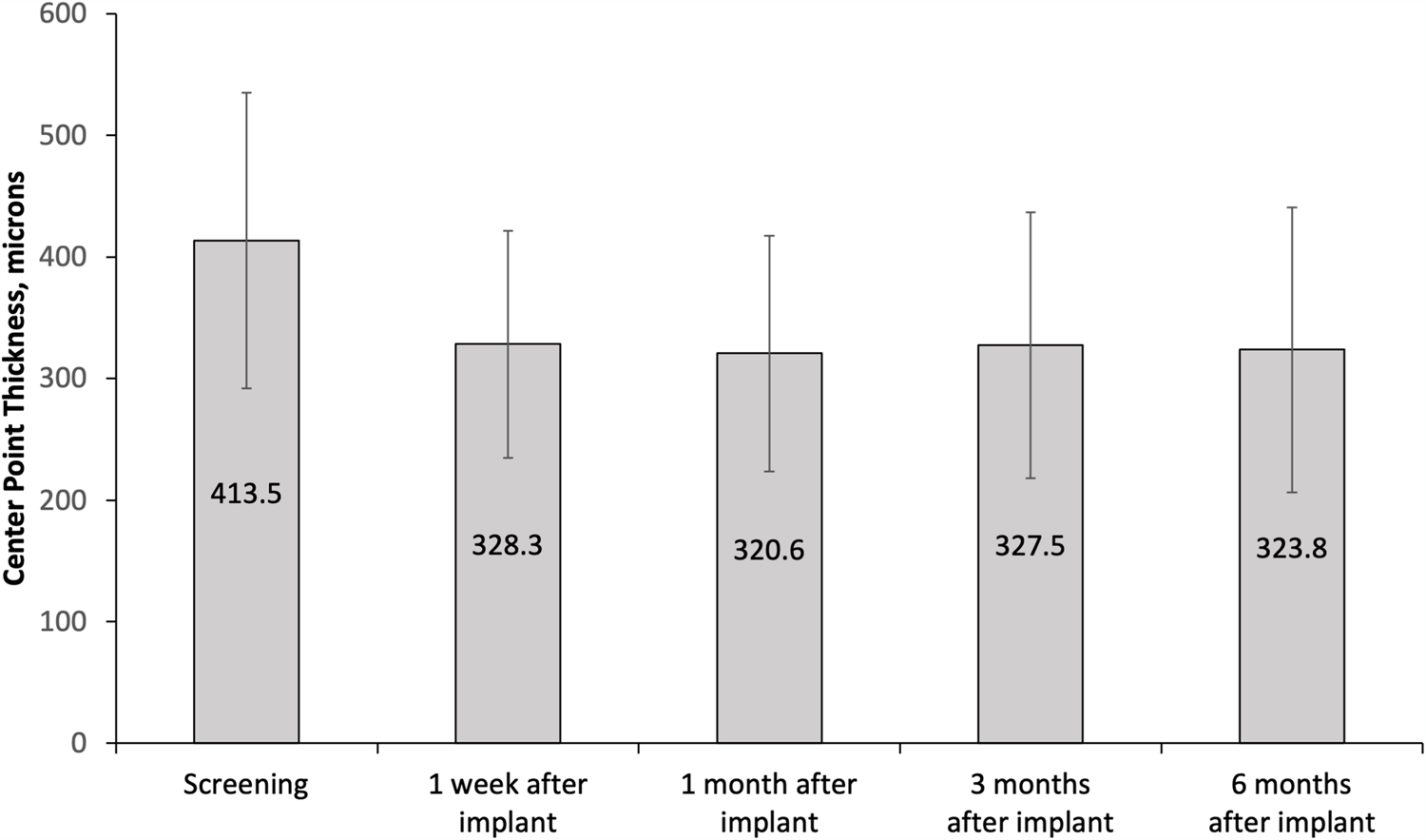
Mean center point thickness recorded at baseline and at time points between 1 week and 6 months after the administration of the FAc implant

The CPT measured during the study is displayed in Fig. 2 and shows a rapid decline by the first follow-up visit (1 week), versus the value recorded at screening (0–4 weeks prior to the administration of the FAc implant), and that this was sustained throughout (*p* < 0.05 for all points versus screening). Table 4 includes individual patient CPT and ART results.

### Intraocular pressure outcomes

**Fig. 3:**
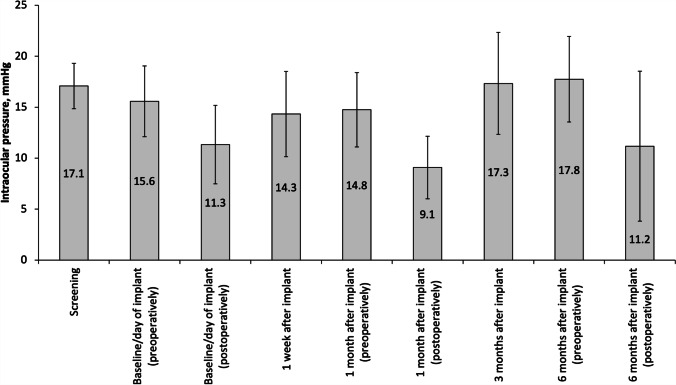
Mean intraocular pressure recorded prior to (screening) and after (1 week to 6 months) the administration of the FAc implant. Notes: Preoperatively refers to the measurement of pressure prior to and postoperatively refers to the measurement of pressure after 2 port pars plana vitrectomy for vitreous probes

Intraocular pressure was measured during the study and mean values are reported in Fig. 3. The lowest mean intraocular pressure (9.1 mmHg) was measured postoperatively at month 1 and the highest mean value (17.8 mmHg) was obtained preoperatively at month 6. There was no significant intraocular pressure change when mean preoperative or non-operative (no probe taken) measurements were compared with mean screening measurements, with the exception of 1 week after implant (*p* < 0.05). For individual results, see Table 5.

**Table 5:** Individual patient intraocular pressure levels (mmHg)

Patient	Screening	Baseline (preoperatively)	Baseline (postoperatively)	1 week after implant	1 month after implant (preoperatively)	1 month after implant (postoperatively)	3 months after implant	6 months after implant (preoperatively)	6 months after implant (postoperatively)
ILV1-01	15	18	4	10	18	12	13	14	7
ILV1-02	17	18	14	19	12	6	21	22	25
ILV1-03	15	14	9	14	14	4	16	18	4
ILV1-04	16	9	10	8	9	11	8	10	4
ILV1-05	17	15	15	10	14	12	15	14	11
ILV1-06	20	23	17	16	16	10	19	22	18
ILV1-07	18	16	14	14	13	9	21	14	7
ILV1-08	13	13	10	12	13	9	23	18	9
ILV1-10	20	18	16	18	13	10	21	18	19
ILV1-11	16	15	8	22	20	7	23	19	20
ILV1-12	20	13	10	17	22	14	18	25	5
ILV1-13	18	15	9	12	13	5	10	19	5
Mean	17.1	15.6	11.3	14.3	14.8	9.1	17.3	17.8	11.2

### Safety

Four events (16.7%) in 3 patients (25.0%) recorded during the study were related to the FAc implant. These were iris adhesions, increased intraocular pressure, a medical device problem (implant was stuck in the needle during application) and a surgery-related problem (extrusion of implant through trocar during vitreous probe insertion). However, none of the adverse events that occurred were serious and all could be resolved without sequelae.

## Discussion

The majority of studies measuring cytokine levels in the eye have sampled fluid from the aqueous humor rather than the vitreous. The reasons for this relate to the fact that obtaining vitreous samples may lead to higher complications compared to samples obtained from the aqueous humor, which are relatively easier and safer to perform. Another reason is that cytokine levels in the aqueous humor have been correlated with levels in the vitreous fluid [25], at least in the case of VEGF and IL-6. However, the same correlation has not been proven for other cytokines and the study by Funatsu et al. [25] did not show a perfect correlation in cytokine levels taken from the 2 different sampling sites in the eye. With this in mind, the current study was designed to assess inflammatory and angiogenic cytokine expression in the vitreous fluid during a partial vitrectomy and in doing so had the advantage of measuring the activity at the site of delivery and sites of action in the posterior segment of the eye.

The current study evaluated the influence of the FAc implant therapy on levels of IL-6 as well as different vitreous inflammatory and angiogenic cytokine levels (including IL-8, IP-10, MCP-1, VEGF, CD54, PIGF and PEDF) in patients with DME. IL-6, as well as MCP-1, VEGF, and CD54, have been reported to be significantly higher in patients with DME than in nondiabetic patients [20]. These findings demonstrate the importance of inflammatory cytokines, and not solely the VEGF cytokine, in the breakdown of the blood retinal barrier and the pathogenesis of DME.

It is documented that patients with diabetes, in contrast to non-diabetics, have elevated levels of aqueous and vitreous IL-6 [26]. In the present study, an average concentration of 84.30 ± 126.81 pg/ml was measured in the vitreous at baseline. At 1 and 6 months after the FAc implant, it decreased significantly to 49.35 ± 57.07 pg/ml and 46.93 (± 79.63) pg/ml, respectively. The result of this reduction of inflammatory parameters in the vitreous cavity is consistent with previous work showing inflammatory molecules such as IL-6 in the aqueous humor can be significantly reduced by intravitreal corticosteroid therapy [26]. Ecker et al. [27] failed to show a correlation between inflammatory parameters in the anterior chamber and the vitreous chamber and others [28] have reported that only a small minority of the proteins in both chambers can be correlated. These findings suggest that vitreous samples at the site of disease manifestation may be necessary to reliably demonstrate a response to intravitreal therapy [27–29].

Song et al. [30] postulated that elevated levels of CD54 play a central role in the progression of DR and the severity of disease. Likewise, Funatsu et al. [20] demonstrated a correlation between DME severity and CD54 levels. In this study, there was a statistically significant decrease in CD54 after FAc therapy.

VEGF levels have also been reported to be elevated in aqueous and vitreous humor in patients with diabetes as compared to non-diabetic patients [26], although there are mixed findings concerning the relationship between VEGF and DR or DME severity [20, 26, 30]. At the beginning of therapy with the FAc implant, this study showed mean VEGF concentrations of 280.31 ± 351.49 pg/ml. After 1 month, the concentration increased slightly to 286.42 ± 337.93 pg/ml and by month 6 had decreased (*P* > 0.05) by 100.71 ± 312.95 pg/ml to a level of 179.61 ± 163.94 pg/ml. The overall tendency was for VEGF to decrease, but this was not statistically significant as VEGF levels increased in 4 eyes (range, 37.0 to 206.3 pg/ml) and remained unchanged in 1 eye.

The previous work of Song et al. [30] and Dong et al. [26] showed that the amount of MCP-1 in aqueous humor or vitreous humor also correlates with the severity of DR or DME. At the beginning of the present study the MCP-1 concentration was 866.46 ± 400.97 pg/ml. At month 1, it had decreased to 545.63 ± 235.58 pg/ml and at month 6 to 541.85 ± 241.04 pg/ml. The decrease in MCP-1 values was statistically significant (*p* = 0.0005) and indicates an effect of the FAc implant on the expression of MCP-1.

IL-8 levels are also associated with the severity of DR [30]. At baseline, the average IL-8 concentration was 39.90 ± 35.33 pg/ml. Slight decreases but non-significant changes were observed at month 1, (to 37.09 ± 26.74 pg/ml) and month 6 (to 33.80 ± 24.97 pg/ml). Therefore, an effect of FAc implant therapy on IL-8 concentration could not be shown.

The mean PIGF concentration before treatment with the FAc implant was 95.86 ± 108.35 pg/ml. At month 1 it increased to 128.49 ± 157.67 pg/ml and at month 6 decreased to 75.79 ± 63.17 pg/ml. Overall, a statistically significant decrease in PIGF concentration of 20.07 ± 67.71 was observed by month 6.

The IP-10 concentration was 134.81 ± 241.71 pg/ml on the day of FAc implantation. A statistically significant decrease in IP-10 concentration was observed during the course of the study. One month after transplantation, the IP-10 concentration was 76.50 ± 125.21 pg/ml. A further decrease (to 64.13 ± 88.91 pg/ml) was observed by month 6. Overall, there was a decrease of 70.68 ± 155.34 pg/ml by month 6.

At baseline the average PEDF concentration was 4696.15 ± 315.84 pg/ml. During the course of the study, the concentration increased slightly at month 1 (4742.98 ± 270.44 pg/ml). At month 6, the mean concentration was 4711.48 ± 266.64 pg/ml, which was similar to the baseline value and only changed by 15.33 ± 439.60 pg/ml (*p* > 0.05).

Regarding clinical results, the FAc implant is a sustained delivery device but still, the decrease in center point thickness occurred mostly in the first month after implantation of the FAc implant. Over the 6-month follow-up period, this decrease was sustained. This is adventitious to patients as they do not have to receive ongoing injections to obtain a similar result [5]. Only 1 patient had a mild steroid response resulting in increased (up to 25 mmHg) intraocular pressure following device implantation. This was resolved with topical antiglaucoma therapy. As seen in Table 5, due to the vitreous probe, there was an induced preliminary hypotony in 7 of 36 postoperative measurements but without clinical complications such as corneal changes or choroidal folds. This preliminary IOP fluctuation might have an impact on the cytokine concentrations due to the potential blood retinal barrier and blood aqueous barrier breakdown [31]. This should be considered as a limitation of the study.

An additional limitation of the current study is that it included a cohort of 12 patients from a single clinical center. Hence, it needs to be confirmed in a larger cohort of patients across multiple centers. Furthermore, the FAc implant has been designed to provide sustained drug delivery for up to 3 years and some parameters (e.g., PIGF and PEDF) only started to decrease by month 6 or decreased slowly over the study period. From clinical data [2] we know that the FAc implant has an effect for up to 3 years but to our knowledge, cytokine levels have not been evaluated past 6 months post implantation up to this point. A single case reported by Singh et al. [32]. showed that edema recurred approximately 3 years after implantation and it is speculated that this was driven by the underlying inflammatory cascade. Variables such as HbA1c which reflect the lifestyle of the patient might also play a role in cytokine changes. A study with a longer study period (i.e., 12 months or longer) with an additional vitreous probe would be valuable. Regarding statistical analysis, the study was designed to investigate whether the intravitreal FAc implant has an influence on IL-6 at baseline and month 1 and 6 (primary parameter). The other cytokines, evaluated at the same time points, were designated as secondary parameters. A further study could consider performing multiple comparisons over time if multiple cytokines are evaluated from the same vitreous samples.

This study reports changes in vitreous inflammatory and angiogenic cytokine levels following intravitreal injection of the FAc implant in patients with DME. Data show that the FAc implant altered the expression of vitreous inflammatory and angiogenic cytokine levels, which were maintained 6 months after administration of a single FAc implant. These effects were also accompanied by improvements in visual acuity and center point thickness.

## Supplementary Information

Below is the link to the electronic supplementary material.

Supplementary file1 (MP4 11455 KB) Supplementary Information 1: Video that demonstrates a limited 2-port pars plana vitrectomy with vitreous probe.

## Data Availability

Data is available on request from the corresponding author.
